# Gastrointestinal Side Effects of Antiarrhythmic Medications: A Review of Current Literature

**DOI:** 10.7759/cureus.1646

**Published:** 2017-09-03

**Authors:** Waseem Amjad, Waqas Qureshi, Ali Farooq, Umair Sohail, Salma Khatoon, Sarah Pervaiz, Pratyusha Narra, Syeda M Hasan, Farman Ali, Aman Ullah, Steven Guttmann

**Affiliations:** 1 Forest Hills Hospital, Northshore-Long Island Jewish Health System; 2 Internal Medicine, Wake Forest Baptist Hospital; 3 Internal Medicine, West Virginia University - Charleston Division; 4 Gastroenterology and Hepatology, East Texas Medical Center; 5 Internal Medicine, Northwell - Long Island Jewish Forest Hills Hospital; 6 Medicine, Northwell - Long Island Jewish Forest Hills Hospital; 7 Medicine, St.john Hospital and Medical Center, Detroit; 8 Internal Medicine, St Joseph Mercy Oakland Hospital; 9 Digestive Diseases, Northwell - Long Island Jewish Forest Hills Hospital

**Keywords:** antiarrhythmic drugs, cardiac drugs, cardiac pharmacology, gastrointestinal side effects, drug side effects

## Abstract

Antiarrhythmic drugs are commonly prescribed cardiac drugs. Due to their receptor mimicry with several of the gastrointestinal tract receptors, they can frequently lead to gastrointestinal side effects. These side effects are the most common reasons for discontinuation of these drugs by the patients. Knowledge of these side effects is important for clinicians that manage antiarrhythmic drugs. This review focuses on the gastrointestinal side effects of these drugs and provides a detailed up-to-date literature review of the side effects of these drugs. The review provides case reports reported in the literature as well as possible mechanisms that lead to gastrointestinal side effects.

## Introduction and background

Antiarrhythmics are commonly prescribed drugs and have a narrow therapeutic window. These medications mainly affect the ion channel in the cardiac cell membrane. They are pro-arrhythmogenic and can also cause systemic side effects including gastrointestinal side effects. Antiarrhythmic drugs have sometimes been discontinued because of these side effects. For example, amiodarone is known for pro-arrhythmogenic side effects but there are instances where the drug is stopped because of liver toxicity. Based on the Vaughan Williams classification of antiarrhythmic drugs, they are classified into four classes depending on the mechanism of action (Table 1) [1]. Class 1 is further classified into a, b, and c. Antiarrhythmics are prescribed based on an individualized approach, not following the Vaughan classification. They are either used as a single drug or in combination with multiple drugs. Some of the antiarrhythmics have more than one class action; for example, amiodarone has all four class effects and sotalol has class 2 and 3 effects. As most antiarrhythmics are metabolized and eliminated by the liver, chronic use or overdose of these drugs can lead to hepatotoxicity.

**Table 1:** Classification of Antiarrhythmic Medications Modified from Kowey, et al. [1]

Classes	Membrane Effect	Drugs
Class 1a	Sodium channel block; intermediate kinetics, and potassium channel block	Quinidine, procainamide hydrochloride, disopyramide
Class 1b	Sodium channel block; rapid kinetics	Lidocaine, tocainide, mexiletine hydrochloride, phenytoin
Class 1c	Sodium channel block with slow kinetics	Flecainide and propafenone
Class 2	Beta-receptor blocker	Propranolol, metoprolol, esmolol, acebutolol, atenolol
Class 3	Potassium channel blocker, slow sodium channel facilitation	Procainamide, sotalol, dronedarone, dofetilide
Class 4	Calcium channel blocker	Verapamil and diltiazem
Digitalis	Sodium potassium ATPase inhibition	Digoxin, digitoxin
Adenosine	Inhibit cyclic adenosine monophosphate (cAMP) mediated calcium influx	Adenosine

## Review

Class 1a

Class 1a drugs include quinidine, disopyramide, and procainamide.

Quinidine

Quinidine is an optical isomer of quinine and is derived from the bark of cinchona. Quinine is used for the treatment of parasitic infections, such as malaria. Quinidine is an older drug and not used frequently because of the development of newer antiarrhythmic drugs.

Studies have shown that quinidine causes hepatotoxicity in 2% to 2.2% population. Cases presented in the literature are mostly of granulomatous hepatitis (Table 2) [2]. Clinical presentation of hepatitis, such as fever, rash, thrombocytopenia, reversibility on discontinuation of the drug, and reappearance after rechallenge, explains quinidine hepatitis as a hypersensitivity reaction. When hepatotoxicity develops, treatment is discontinuation of the medication.

**Table 3:** Summary and Comparison of a Few Cases of Quinidine-induced Hepatotoxicity

References	Age (Years)/ Sex	Symptoms, signs and labs	Duration of Drug Use	Outcome	
					Repeat Exposure
Guharoy, et al. [3]	62/Male	Diarrhea, nausea, fever, palpitations, elevated aspartate aminotransferase, and alanine aminotransferase	Two weeks	Symptoms resolved in 42 hours, liver enzymes started decreasing gradually and normalized on day 12.	No
Bramlet, et al. [2]	Unknown	Fever, urticaria, and mild thrombocytopenia	Unknown	Histology showed granulomatous hepatitis. Symptoms resolved with discontinuation of the drug.	Symptoms appeared three days after readministration
Handler, et al. [4]	72/Female	Anorexia, weight loss, elevated aspartate aminotransferase, alkaline phosphatase, and lactate dehydrogenase	16 months	Active hepatitis on liver biopsy, liver functions normalized after discontinuation of the drug.	Re-challenge with the drug caused elevation of liver enzymes and lactate dehydrogenase
Hogan, et al. [5]	85/Male	Jaundice and vomiting, elevated aspartate aminotransferase, alkaline phosphatase, lactate dehydrogenase, and total bilirubin	8 days	Biopsy showed centrilobular cholestasis and granulomatous hepatitis. Quinidine stopped on day 10 and liver functions started improving.	No

As a result of its prolonged transit time, quinidine has also been reported to cause esophagitis. On average, most cases are seen at the age of 60; esophagitis caused by quinidine can lead to strictures. The condition can be prevented by taking pills in sitting position and with plenty of water. Acid suppression with histamine H2 blockers, sucralfate, and proton pump inhibitors are commonly used but their efficacy has not been clearly established. In cases where complications like strictures and perforation develop, surgical intervention may be needed [6]. Quinidine rarely causes pill-induced gastritis; however, one case of gastric ulceration has been reported. The case was managed with discontinuation of drug and use of sucralfate and rabeprazole [7].

Disopyramide

Disopyramide has similar electrophysiological properties as quinidine with fewer gastrointestinal side effects. It is mainly used for the treatment of ventricular tachycardia.

Due to anticholinergic action, disopyramide can cause constipation and dry mouth, which can be controlled by the acetylcholinesterase inhibitor, pyridostigmine. Studies show that pyridostigmine has no significant effect on antiarrhythmic properties of the drug. Although disopyramide has fewer gastrointestinal side effects, cases of hepatotoxicity have been reported, as well as hepatic ischemia secondary to cardiac failure by passive congestion. The drug has a negative ionotropic effect which can worsen heart failure. Liver functions and symptoms improved after the discontinuation of the drug, and in one case, diuretics were used to resolve congestion [8]. Rarely, disopyramide can cause cholestatic jaundice that, in most cases, appears within the first week of taking the medication. Generally, cholestatic jaundice resolves promptly on discontinuation of the drug (Table 3) [9]. A case of severe hepatocellular toxicity with disseminated intravascular coagulation was reported, which improved after discontinuation of disopyramide [10].

**Table 2:** Comparison of Cases of Disopyramide-induced Hepatotoxicity

References	Age (Years)/ Sex	Symptom, signs and labs	Duration of drug use	Outcome	
					Repeat Exposure
Scheinman, et al. [8]	62/Male	Anxiety, dyspnea, abdomen pain, elevated aspartate aminotransferase, and lactate dehydrogenase	One day	Hepatic necrosis with congestion on biopsy, disopyramide was discontinued and liver enzymes and symptoms improved on day six.	Disopyramide was restarted but the patient again had a recurrence of anxiety and dyspnea, it was discontinued. Liver enzymes did not elevate. Antiarrhythmic was changed to procainamide.
Scheinman, et al. [8]	61/Male	Hypotension, chest pain, 1+ edema lower extremity, bilateral lung bases rales, hepatomegaly. Aspartate aminotransferase, alanine aminotransferase, and lactate dehydrogenase were elevated	One day	Symptoms improved with diuresis and liver enzymes normalized.	Nausea, dyspnea, and hypotension developed one day after readministration of disopyramide. Liver enzymes peaked; the drug was switched to procainamide and liver enzymes normalized in few days.
Doody [10]	55/Female	Elevated liver enzymes and disseminated intravascular coagulation (prolonged prothrombin time, elevated fibrin split products)	Unknown	Both liver enzymes and liver functions improved 14 days after discontinuation of therapy.	No
Barkins, et al. [9]	Unknown	Jaundice and abnormal liver enzymes	One week	Prompt clinical resolution on discontinuation of the drug.	No

Procainamide

Procainamide is frequently used for atrial and ventricular arrhythmias. Although rare, procainamide can cause hepatotoxicity and cases have been reported of granulomatous and intrahepatic cholestasis. Hepatotoxicity caused by procainamide is due to an unknown mechanism, but most likely it is secondary to hypersensitivity. Clinical features of hypersensitivity, including fever, rash, and nausea, are usually present. In most cases, liver function improves in days to weeks on discontinuation of the drug. There is only one case reported of acute pancreatitis secondary to procainamide drug-induced lupus [11]. Due to anticholinergic action, procainamide can cause constipation. The literature has only one report of a case of pseudo-obstruction in a diabetic patient with procainamide, which can happen with both oral and intravenous forms of the drug. The condition improved with the discontinuation of the drug [12].

Class 1b

Class 1b drugs include lidocaine, phenytoin, mexiletine, and tocainide. Lidocaine is not reported with significant gastrointestinal side effects.

Phenytoin

Phenytoin is derived from hydantoin and is commonly used for seizure treatment. As the drug has an effect on ion channels of the cardiac membrane, it can be used as antiarrhythmic. Phenytoin is mainly metabolized and eliminated by the liver and has a narrow therapeutic window.

Phenytoin can rarely cause both acute and chronic pancreatitis. A proposed mechanism for chronic pancreatitis is the chronic induction of P450 and usually develops over years. Acute pancreatitis usually resolves with discontinuation of the drug [13].

Phenytoin is a liver enzyme inducer and can affect the metabolism of other drugs. Phenytoin can cause both acute and chronic liver damage. Liver injury can be cholestatic, cytotoxic, or mixed. Literature shows hypersensitivity is a cause of liver toxicity in over 70% cases. It can also cause irreversible liver damage associated with necrosis. A proposed mechanism is direct cytotoxic effects of toxic metabolites. Phenytoin is an aromatic antiepileptic drug and the drug metabolites cause oxidative stress, which results in hepatotoxicity [14]. There is no defined therapy, and the drug should be discontinued. Use of N-acetylcysteine is recommended in cases with severe liver damage. Also, patients with severe hepatic damage with coagulopathy should be referred to liver transplant centers. It can worsen the hepatotoxic effects caused by acetaminophen overdose (Table 4) [15].

**Table 4:** Comparison of Cases of Phenytoin-induced Hepatotoxicity

References	Age (Years)/ Sex	Symptoms, signs and labs	Duration of Drug Use	Outcome	
					Repeat Exposure
Roy, et al. [16]	52/Female	Elevated serum aminotransferases	11 years	Liver biopsy showed chronic persistent hepatitis. Liver functions improved after discontinuation of the drug.	Etiology confirmed by rechallenge
Brackett and Bloch [17]	55/Female	Elevated liver enzymes	Unknown	Liver enzymes improved after discontinuation of acetaminophen. Phenytoin predisposed acetaminophen toxicity	Phenytoin continued.
Suchin, et al. [15]	22/Male	Hepatic encephalopathy, acute renal failure, elevated liver enzymes	Unknown	Hemodialysis initiated and liver transplantation done, explant showed hepatic necrosis. Phenytoin worsened the acetaminophen-induced hepatotoxicity	Unknown
Altuntas, et al. [18]	47/Female	Exfoliative dermatitis, increase in liver enzymes with a cholestatic pattern, eosinophilia	25 days	Liver biopsy consistent with drug-induced hepatitis; liver functions improved three weeks after discontinuation of phenytoin	No

Mexiletine and Tocainide

Mexiletine have has the similar structure as lidocaine and acts with the similar mechanism. At the beginning, it was used as an analgesic and is now proved effective for management of ventricular arrhythmias. Mexiletine is reported to cause esophagitis and esophageal ulcers. Cases with esophageal ulcers are treated with acid suppression. Prevention is recommended, specifically, taking the drug in the upright position with a significant amount of water [19].

Tocainide is a lidocaine analogue and has a similar electrophysiological effect. Tocainide can rarely cause hepatitis; a case with granulomatous hepatitis has been reported [20].

Class 1c

Class 1c drugs include flecainide and propafenone.

Flecainide and Popafenone

Flecainide is used for supraventricular arrhythmias as atrial fibrillation. Flecainide rarely causes gastrointestinal side effects. A case of hepatitis caused by flecainide has been reported. The assumed mechanism of hepatotoxicity was an allergic reaction, and the liver enzymes improved after withdrawal of drug [21].

Propafenone has a beta blocker effect and can cause bradycardia. It is primarily metabolized by the liver. There are a handful of cases of propafenone-induced hepatotoxicity; toxicity can be hepatocellular or cholestatic. Liver functions improve with discontinuation of the drug [22].

Class 2

Class 2 drugs include beta blockers, such as propranolol, metoprolol, atenolol, esmolol, and acebutolol. 

Beta-adrenergic Receptor Blockers

Beta blockers are commonly used for hypertension, arrhythmias, migraine, glaucoma, and anxiety. Beta blockers decrease mortality in patients with atrial fibrillation with or without heart failure. Selective beta-adrenergic blockers are used for atrial and ventricular arrhythmias. Beta blockers have a wide therapeutic index but their use is associated with various side effects.

Although very rare, atenolol, carvedilol, and metoprolol can cause hepatotoxicity as evident in case reports but the etiology is unknown. Atenolol-associated hepatotoxicity is observed in very few cases, liver biopsy shows inflammatory infiltrate suggestive of immune-mediated process and cases improved with discontinuation of the drug (Table 5) [23]. The study by Packer, et al. showed that 0.3% of patients of carvedilol developed abnormal liver functions leading to discontinuation of drug [24]. There is only one case of metoprolol-induced hepatotoxicity. A proposed mechanism was poor oxidation of the drugs debrisoquine and perhexiline [25].

**Table 5:** Comparison of beta blocker hepatotoxicity cases

Reference	Drug	Age (Years)/ Sex	Symptoms, signs and labs	Outcome	
					Repeat Exposure
Kootte, et al. [26]	Sotalol	68/Female	Elevated Liver enzymes	Liver functions Normalized in five months after discontinuation of the drug.	Unknown
Hagmeyer and Stein [27]	Carvedilol	40/Male	Pruritis, elevated serum transaminases	Carvedilol was discontinued, symptoms improved with hydroxyzine. The drug was discontinued and liver enzymes improved after three weeks.	One year later, the patient was started on metoprolol and developed pruritus in 10 days and the drug was stopped. Liver enzymes remained normal.
Dumortier, et al. [23]	Atenolol	57/Female	Asthenia and elevated liver enzymes	Liver biopsy showed portal and centrilobular inflammation. After receiving steroids, liver function tests improved but were still elevated; repeat biopsy showed necrosis in centrilobular areas with pigmented macrophages. Liver function tests normalized two months after discontinuation of atenolol. Control liver biopsy in seven months showed disappearance of centrilobular lesions.	No

Propranolol can be a risk factor for ischemic colitis if a patient is on multiple antihypertensive medications. There is a case where the patient was on nicardipine hydrochloride, propranolol hydrochloride, and digoxin for hypertension; she developed ischemic colitis post-colonoscopy and clinically improved with conservative management [28].

Class 3

Class 3 drugs include amiodarone, sotalol, dronedarone, and dofetilide.

Amiodarone

Amiodarone has a similar structure as thyroid hormone. It is a lipophilic drug and can deposit in fat tissues and liver. It is metabolized slowly, so its effects take longer to resolve. As discussed earlier, amiodarone has multiple class actions. Long-term use can cause extracardiac adverse effects as pulmonary toxicity, thyroid dysfunction, corneal deposits, and liver toxicity [29].

Hepatotoxicity ranges from asymptomatic elevated transaminases (25%) to severe liver damage (1% - 3%). Asymptomatic abnormal liver function tests (LFT) usually resolve with dose reduction or stopping the drug. Liver toxicity has been observed with both oral and IV drugs. The mechanism of liver damage with amiodarone is unclear. It could be immune-mediated hepatocyte damage with free radical formation. Damage secondary to parenteral amiodarone may be due to solvent Polysorbate 80. Polysorbate 80 is short-acting and liver function improves quickly after discontinuation of parenteral amiodarone [29]. In patients where hepatotoxicity due to amiodarone infusion is seen, oral amiodarone is safe to continue (Table 6). The North American Society of Pacing and Electrophysiology (NASPE) recommends the monitoring of LFT every six months after a baseline check at the time of starting the amiodarone [30].

**Table 6:** Comparison of a Few Case Reports of Amiodarone-induced Hepatitis in the Literature

References	Age (Years)/ Sex	Symptoms, Signs and Labs	Duration	Outcome	
					Repeat Exposure
Singhal, et al. [31]	79/Male	Coffee ground emesis, lethargy, ascites, spider nevi, gynecomastia, palmar erythema	33 months	Cirrhosis and extensive fibrosis confirmed on liver biopsy. Amiodarone was discontinued. The patient developed renal failure, heart failure, hepatic encephalopathy and died in three months.	No
Rizzioli, et al. [32]	79/Female	Liver function tests increased acutely after the start of amiodarone infusion	One day	Amiodarone infusion was stopped, transaminases started to decrease. Progressive congestive heart failure led to death on same admission.	No
Kang, et al. [33]	75/Male	Nausea, vomiting, and polyneuropathy for three months and mildly elevated liver enzymes	17.8 months	Liver biopsy showed microvesicular steatosis, foam cells, and Mallory bodies. Amiodarone was discontinued; liver enzymes improved on day three and symptoms slowly improved.	No
Lahbabi, et al. [34]	29/Female	Significantly elevated enzymes acutely after starting amiodarone infusion	24 hours	Amiodarone infusion was stopped and replaced by oral amiodarone. Liver functions improved gradually.	Oral amiodarone continued; liver function tests after two months were normal
Fonesca, et al. [29]	88/Male	Significantly elevated liver enzymes after start of amiodarone infusion with thrombocytopenia and acute kidney injury	18 hours	Amiodarone infusion was stopped; liver and renal functions gradually normalized.	Oral amiodarone was restarted on day four at lower dose and liver functions were normal on follow-up
Chen and Wu [35]	Unknown	Acutely elevated liver enzymes after starting amiodarone infusion	24 hours	Amiodarone infusion was stopped; liver enzymes normalized.	Oral amiodarone was started on lower dose and hepatotoxicity did not occur on follow-up

A large retrospective cohort study conducted in Taiwan showed that use of amiodarone is associated with malignant neoplasm of the liver and intrahepatic bile ducts (MNLIHD) in a dose-dependent manner. Malignancy is common in cases with comorbidities, such as diabetes, chronic liver disease, cirrhosis, hepatitis C, hepatitis B, and alcoholism (Table 7) [36].

**Table 7:** Odds Ratio with Confidence Interval of 95% for Malignant Neoplasm of the Liver and Intrahepatic Bile Ducts (MNLIHD) Associated with Amiodarone and Other Antiarrhythmics Modified from Yun-Ping, et al. [36]

Medications	MNLIHD odds ratio (confidence interval 95%)
Amiodarone	1.6 (1.45 - 1.77)
Mexiletine	1.07 (0.98 - 1.18)
Propafenone	0.97 (0.85 - 1.11)
Quinidine	1.32 (0.91 - 1.93)
Procainamide	0.97 (0.71 - 1.33)

Amiodarone can rarely cause acute pancreatitis as there are few case reports in the literature. A case control population study by Lai, et al. showed the current use of amiodarone increases the risk of acute pancreatitis (Table 8) [37]. The exact mechanism of pancreatitis caused by amiodarone is unknown. Proposed mechanisms are direct toxicity caused by amiodarone and its metabolite, mono-N-desethylamiodarone, due to its lipophilic nature. Amiodarone is metabolized by P450, and pancreatitis can be caused by drug-drug interaction. The case reports show improvement of pancreatitis symptoms with discontinuation of the drug (Table 9).

**Table 8:** Amiodarone-associated Acute Pancreatitis Modified from Lai, et al. [37]

Amiodarone use	Acute pancreatitis adjusted odds ratio (confidence interval 95%)
Current Use	5.21 (3.22 - 8.43)
Recent Use	1.18 (0.32 - 4.35)
Past Use	1.42 (0.99 - 2.03)

**Table 9:** Summary and Comparison of Cases of Amiodarone-induced Pancreatitis

References	Age (Years)/ Sex	Symptoms, signs and labs	Duration	Outcome	
					Repeat Exposure
Bosch and Bernadich [38]	46/Female	Nausea, vomiting, epigastric pain, elevated lipase	Four days	Intravenous fluids were given; symptoms resolved after discontinuation of the drug. Procainamide was started.	Procainamide was stopped and amiodarone was started at low dose 100 mg per day. After three days, the patient had recurrence of symptoms with elevated amylase and lipase. Amiodarone was stopped and procainamide was restarted.
Famularo, et al. [39]	80/Male	Epigastric pain, vomiting, distended abdomen, elevated amylase, and lipase	Five days	Amiodarone dose was reduced to 200 mg daily from loading dose; symptoms and serum lipase levels improved after three days.	Rechallenge with a high dose of amiodarone was not performed and continued with 200 mg.
Chen, et al. [40]	66/Female	Epigastic pain, loss of appetite, and elevated lipase	Three months	Abdominal pain persisted even after three weeks of conservative management. All causes ruled out. Amiodarone was substituted with propafenone; symptoms resolved and lipase gradually trended down	No

Sotalol

Sotalol is a non-selective beta blocker and can be used for the treatment of atrial fibrillation, ventricular tachycardia, premature ventricular contractions, and supraventricular tachycardia. Sotalol is a hydrophilic drug and not metabolized by the liver; still, one case of severe chronic hepatitis has been reported. The mechanism of liver injury is unknown. Patient symptoms improved and liver functions normalized on discontinuation of the drug (Table 5) [26].

Dronedarone

Dronedarone is a newer drug and has a structure similar to amiodarone. The drug is used for atrial fibrillation and atrial flutter. Dronedarone can cause hepatotoxicity with mitochondrial damage. A study on rat liver cells explained the mechanism, as it inhibited the electron transport chain and beta oxidation; this caused increased production of reactive oxygen species (ROS), which led to apoptosis and mitochondrial damage [41]. There are a few cases of severe toxic hepatitis. Two of the hepatic failure cases were treated with the emergent liver transplant (Table 10) [42].

**Table 10:** Summary of Cases of Dronedarone-induced Hepatotoxicity ICU: intensive care unit

Reference	Age (Years)/ Sex	Symptoms, Signs and Labs	Duration	Outcome	
					Repeat Exposure
Del Pozo Ruis, et al. [43]	75/Male	Nausea, vomiting, abdominal pain, elevated liver enzymes	Four days	Patient developed fulminant liver failure, dronedarone was discontinued. Liver functions improved after seven days of discontinuation and ICU monitoring	No
Jahn, et al. [42]	69/Female	Elevated liver enzymes	Unknown	Acute liver failure resolved after discontinuation of the drug.	Unknown
Rizkallah, et al. [44]	85/Male	Nausea and elevated liver enzymes	One day	Drug was discontinued after three doses; the patient developed multiorgan failure and died on day 8.	No

Class 4

Class 4 drugs include calcium channel blockers (CCB), such as verapamil and diltiazem.

Verapamil

Verapamil is commonly used for supraventricular tachycardia, hypertension, and angina. Verapamil not only acts on the calcium channel in the heart muscle but also on vascular and gastrointestinal smooth muscle. The most common adverse effect of verapamil is constipation ranging from 6-8%. The mechanism is likely slow transit time in the colon and less likely upper gastrointestinal tract. Constipation is sometimes significant enough to discontinue the drug. Verapamil was also reported to aggravate a pseudo-obstruction in a case report, which improved once verapamil was discontinued [45].

A prospective cohort study showed that CCBs used for hypertension, including verapamil and diltiazem, increased the risk of gastrointestinal hemorrhage in the elderly population. Therefore, CCBs need to be used with caution in an elderly population with risks of gastrointestinal hemorrhage [46]. Rarely, verapamil can lead to idiosyncratic hepatitis. Symptoms and liver functions improve with discontinuation of drug [47].

Diltiazem

Diltiazem is commonly used for supraventricular tachycardia, hypertension, and angina. The drug has an extensive first-pass hepatic metabolism. CCBs are a weak inhibitor of CYP 3A4 and increase serum levels of medications metabolized by CYP 3A4. Intravenous infusion of diltiazem can drop the blood pressure and can lead to hypoxic liver injury. There was a case of hypoxic hepatitis in a patient who was started on diltiazem for atrial fibrillation. Liver congestion due to heart failure can also be a contributing factor. In this case of hypoxic hepatitis, the diltiazem infusion was stopped; anticoagulation was held initially because of liver toxicity-induced coagulopathy [48].

A case of paralytic ileus was reported in a patient taking diltiazem for atrial fibrillation and rapidly improved after discontinuation of the drug. The proposed mechanism was decreased gastrointestinal motility due to inhibition of calcium channels in GI smooth muscles [49].

Digoxin

Digoxin is one of most commonly used antiarrhythmics. It is also used in patients with atrial fibrillation and heart failure. Digoxin can cause nonspecific gastrointestinal side effects, such as nausea and vomiting.

A case control study by Lai, et al. showed an increased odds ratio of acute pancreatitis in patients with active digoxin use, and risk was further increased in a patient with chronic kidney disease as 70% of digoxin is normally excreted from the kidneys. The Food and Drug Administration (FDA) has reported 0.2% of cases of acute pancreatitis from 1997 to 2012 in patients on digoxin. The exact pathophysiology was not known; it could have been due to a drug-drug interaction. The proposed mechanism was that digoxin causes hypomagnesemia, which leads to increased glycosaminoglycan levels in the pancreas and can lead to pancreatitis [50].

Adenosine

Adenosine is a natural nucleoside, is given as an intravenous infusion, and has a very short half-life. It is used for the treatment of paroxysmal supraventricular tachycardia. Because of its short half-life, adenosine is not associated with any major adverse effects.

## Conclusions

In conclusion, the gastrointestinal side effects of anti-arrhythmic drugs could lead to discontinuation of these medications in several appropriately treated patients. Knowledge of these side effects could help in identifying these side effects and appropriate drug therapy in patients with arrhythmia.
